# Letter from Accoucheur. No. 3

**Published:** 1845-12-01

**Authors:** 


### Letter from Accoucheur.—No. 3.

Communicated for the Buffalo Medical Journal.

An experienced Accoucheur will seldom fail to discover the actual presentation of the fœtus in the commencement of labor, and it is proper that examination be made early, *in every case,* for that purpose. If it be ascertained that the presentation is a natural one, and there are no untoward symptoms, the accoucheur will not only feel at ease himself, but he will have pleasure in relieving the anxiety of the parturient woman. In such cases, all interference is mischievous, if not positively injurious, until the period arrives when the operator can afford protection to the patient. Many of the accidents of the parturient state arise, it is not to be doubted, from treating it as a state of *disease*. The young accoucheur boasts of having perpetrated an accouchment—of having achieved the extraordinary affair in an inconceivably short space of time; or, that he had labored hours, without intermission or cessation, before he could accomcomplish it. In the first case, the labor would have terminated, probably, within the time, without his assistance; and in the latter, much soorer, no doubt, but for his officious intermedling. On the other hand, if it be ascertained that the presentation is an unnatural one, the accoucheur may have time to prepare himself for the emergency, and to prepare the patient, also, to submit to necessity. Concealment, generally, is as unnecessary, as it is unworthy of the accoucheur. A sensible woman will seldom be reluctant to submit to whatever a frank, confiding physician may think necessary to require. But there are situations and conditions of great embarrassment, perplexity, and anxiety, where doubts exist, and symptoms demand immediate decision, if not instant action—where deliberation is death. No man can even contemplate unmoved, a being placed in a position where life or death depends on instant decision and action, and he alone responsible for the result. How then will he bear up when he finds himself suddenly, and unexpectedly involved in the dreaded dilemma! Nothing can occur in the puerperal state more fearful and appalling than hæmorrhage, or convulsions, before and after delivery. Some cases of the latter, before delivery, will be the subject of this paper.

Mrs. ——, a young woman, in labor with her first child, fell into convulsions at a time when the accoucheur was momentarily expecting the expulsion of the child. Blood was freely abstracted, but the convulsions continuing, two other medical gentlemen were called in, one of whom had the reputation of using the forceps with dexterity and tact. Convulsions commenced at mid-day, and when I saw the patient at 8 o'clock the evening after, she was insensible, cold, pulseless at the wrist, and seemed to be in the article of death. It was reprepresented to have been impracticable to use the forceps, as every attempt to apply them produced a strong convulsion. Convulsions had now ceased, and with one blade of the forceps she was instantly,as it were, delivered of a stout, living child. The Placenta was soon removed, the patient put into bed, and by the assiduous use of warm appliances, in an hour or two she began to rally; the pulse returned to the wrist, but it was twenty-four hours before she was restored to consciousness. Her recovery was speedy, and she has borne children since without accident.

Mrs. ——, a young woman, was taken in labor with her first child, and immediately fell into strong convulsions. The patient was florid and sanguineous. Blood was freely drawn from the arm, and the bowels were evacuated by artificial means. Convulsions continuing, nevertheless, unabated, I saw the patient about four hours after the attack. It happened that both the physician in attendance and myself were unprovided with instruments, and we were both, also, some miles from our respective homes. It was decided, therefore, while I remained with the patient, my friend should go for his instruments. In this interval the friends of the patient were importunate, clamorous for "something to be done."—To quiet them I sat by the patient, and ascertained that the presentation was natural, the waters discharged, the os tincæ dilated to the size of a shilling, and its lips thin. I noticed, too, that whenever the uterus began to contract, the pain was lost in a strong, general convulsion. It occurred to me that the pains might be concentrated by irritating the os uteri, passing a finger around the tincæ and making gentle attempts at dilatation whenever the uterus began to contract, and I was gratified to notice, that though the convulsions continued, the patient was provoked by the irritation to make some expulsive efforts, and the labor was progressing. Without leaving my seat, having ordered a wet napkin around the head, the manipulations were continued a full hour and a half, when my friend returned with his instruments, just as the mother gave birth to a healthy, living child by natural efforts. The placenta came away in due time, the convulsions ceased, and the mother was put into bed. She was restored to consciousness after some hours, recovered speedily, and has since borne children without accident.

Mrs. ——, a young woman, met with a fall a few days before her expected parturition. At full period labor commenced, and went on without accident or interruption some hours, when, without ostensible cause, she fell into convulsions. Though the attending physician was familiar with the practice described in the foregoing case, he invited me to join him in attendance on this, and the practice before described, was adopted in this case, and we sat, alternately, between four and five hours, when the child was expelled, dead, apparently for some days. The placenta came away readily, the convulsions ceased, and in a few hours she was restored to consciousness, and recovered rapidly.

Irritating the os uteri to provoke expulsive pains has been practiced by myself, and many of my friends, for some years, not only in convulsions, but in hæmorrhage, in cases of retained placenta, in abortions and miscarriages—in all cases, indeed, when strong uterine contraction was desired. If there be nothing novel in the practice, these cases furnish additional evidence of its propriety and use.

Puerperal convulsions may arise from irritation, often assuming neuralgic symptoms, simulating hysteria; from cerebral congestion, caused, or aggravated by propulsion of blood to the head in the strong throes of labor; from extravasation of blood on the brain, constituting sanguineous apoplexy. The pathology and treatment of these several conditions do not come within the purpose of this paper, and, perhaps, nothing can be added to what has been already written on the subject.

